# Exploring the influence of human mobility factors and spread prediction on early COVID-19 in the USA

**DOI:** 10.1186/s12889-021-10682-3

**Published:** 2021-03-29

**Authors:** Zhicheng Zheng, Zhixiang Xie, Yaochen Qin, Kun Wang, Yan Yu, Pinde Fu

**Affiliations:** 1 Key Laboratory of Geospatial Technology for Middle and Lower Yellow River Region / College of Environment and Planning Henan University, Jinming Road, Kaifeng, 475004 China; 2 College of Surveying and Geo-informatics, North China University of Water Resources and Electric Power, Zhengzhou, 450046 China; 3 Key Research Institute of Yellow River Civilization and Sustainable Development & Collaborative Innovation Center on Yellow River Civilization jointly built by Henan Province and Ministry of Education, Henan University, Kaifeng, 475001 China

**Keywords:** Human mobility, COVID-19, SIRD model, Prediction, Risk factors, USA

## Abstract

**Background:**

COVID-19 is still spreading rapidly around the world. In this context, how to accurately predict the turning point, duration and final scale of the epidemic in different countries, regions or cities is key to enabling decision makers and public health departments to formulate intervention measures and deploy resources.

**Methods:**

Based on COVID-19 surveillance data and human mobility data, this study predicts the epidemic trends of national and state regional administrative units in the United States from July 27, 2020, to January 22, 2021, by constructing a SIRD model considering the factors of “lockdown” and “riot”.

**Results:**

(1) The spread of the epidemic in the USA has the characteristics of geographical proximity. (2) During the lockdown period, there was a strong correlation between the number of COVID-19 infected cases and residents’ activities in recreational areas such as parks. (3) The turning point (the point of time in which active infected cases peak) of the early epidemic in the USA was predicted to occur in September. (4) Among the 10 states experiencing the most severe epidemic, New York, New Jersey, Massachusetts, Texas, Illinois, Pennsylvania and California are all predicted to meet the turning point in a concentrated period from July to September, while the turning point in Georgia is forecast to occur in December. No turning points in Florida and Arizona were foreseen for the forecast period, with the number of infected cases still set to be growing rapidly.

**Conclusions:**

The model was found accurately to predict the future trend of the epidemic and can be applied to other countries. It is worth noting that in the early stage there is no vaccine or approved pharmaceutical intervention for this disease, making the fight against the pandemic reliant on non-pharmaceutical interventions. Therefore, reducing mobility, focusing on personal protection and increasing social distance remain still the most effective measures to date.

## Introduction

At the end of 2019, a sudden COVID-19 epidemic began to spread rapidly around the world, posing a serious threat to the life and health of residents in many countries, and the sustainable development of both the economy and society [1]. The World Health Organization (WHO) declared COVID-19 a global pandemic on March 11, 2020 [2]. As of 18:00 on May 25th, a total of 216 countries and regions in the world had reported COVID-19 outbreaks. According to COVID-19 epidemic statistics released by Johns Hopkins University (JHU) in the USA on July 26, 2020, as of 02:40 EDT (14:40 Beijing time) on the 26th, the total number of infected cases of COVID-19 in the world had reached 16,048,100, with a total of 644,537 deaths. The USA is the country experiencing the worst onslaught of the epidemic in the world, with a total of 4,178,027 infected cases and 146,460 deaths (https://www.sohu.com/a/409888711_120268273). With the continuous spread of the COVID-19 epidemic, many countries or regions have been forced to take a series of temporary response measures, such as lockdown, suspending business, suspending schools and restricting the movement of people, incurring significant disruptions to the normal operations of social order [3, 4]. In this context, how to accurately predict the turning point, duration and final scale of the epidemic in different countries, regions or cities is very important in enabling decision makers and public health departments to formulate intervention measures and deploy resources [5, 6]. At the same time, it is also a scientific problem requiring an urgent solution.

The SIR model, which originated from epidemic research, is a commonly used dynamic model of infectious diseases. The model was first proposed by Kermack and McKendrick to abstractly describe the process of disease transmission and predict its development trend [7]. The SIR model and its modified versions have been widely applied to the current outbreak of COVID-19 and yielded fruitful research results, which are of great guiding significance for the prevention and control of the epidemic. For example, based on COVID-19 infected cases in China and data pertaining to residents’ travel (including by train, plane and cars), Joseph et al. (2020) estimated the scale of the epidemic with the help of mathematical modeling, and concluded that approximately 75,815 people had been infected in Wuhan, China, during the early stage of the outbreak (i.e., from December 1, 2020 to January 25, 2020) [8]. Fanelli et al. (2020) based on data relating to cumulative infected, recovered and fatal cases of COVID-19 in China, Italy and France from January 22 to March 11, 2020, predicted trends of the epidemic in these countries by building a SIRD model [9]. The results showed that the peak of the epidemic in Italy occurred on March 21 (15,000 new infections in a single day), and that the epidemic would lead to 9300 deaths. In addition, the study also revealed that future national COVID-19 infections in the above-mentioned countries would account for the proportion of the total number of 10–20%, and that the COVID-19 death rate in Italy would remain between 3 and 7%, while the death rate in China would only be 1–3%. Jonathan et al. (2020) used the SEIR transmission dynamics model to simulate the impact of travel restrictions on the development of the epidemic in Wuhan, China [10]. They argued that the “lockdown” in Wuhan on January 23 reduced the spread intensity of the epidemic by 72–75%, expecting the number of new infections in Wuhan to peak on February 4. Based on the cumulative data of infected and cured cases of COVID-19 issued by Chinese health committees at all levels from January 23 to February 1, 2020, Yan et al. (2020) employed a time-delay dynamic model to predict the trend of the epidemic [6]. They claimed that the epidemic could be controlled in mid-late February if prevention and control efforts were kept unabated. Yu et al. (2020) predicted the trend of the epidemic in China using the SIR model and found that the intervention measures taken by the government reduced the actual number of infections by 1/2 compared with the estimated number [11]. Based on the epidemic data released by Chinese officials from January 25 to February 22, and with the help of an improved SEIR model to simulate the epidemic trend [12], Lin argued that China would usher in the “turning point” of the epidemic in mid-March, that residents would essentially be able to return to normal production and life by the end of April, and that the cumulative number of COVID-19 infected cases would remain at about 100,000.

A review of the literature found that research by both current domestic and foreign scholars regarding the COVID-19 pandemic has the following deficiencies. First of all, from the disciplinary point of view, most existing studies explore the gene sequence, source, intermediate host and key factors of COVID-19 virus transmission from the perspectives of pathology, epidemiology, genomics and clinical medicine. They then analyze the potential harm, transmission path and risk factors of COVID-19 epidemic through these factors [13–15]. Although some scholars try to predict the development trend of the epidemic in the future with the help of traditional econometric analysis methods or by constructing mathematical models, there exist certain limitations, such as too short time series and low goodness of fit [9]. Second, from the perspective of spatial scale, scholars have focused on the epidemic trend of COVID-19 in a city or an area of larger scale in China, but with few comparative studies on the epidemic trend in other countries or regions [11, 12, 16]. In fact, China’s quick and effective epidemic control measures have greatly reduced the transmission rate, which will inevitably cause an outbreak with different degrees of deviation between the traditional epidemic trend prediction model and the actual situation.

Third, the effect of “social distance” on the infection rate should be taken into account in prediction models [17, 18]. Early studies have shown that human mobility has played an important role in the dispersal of infection, given that COVID-19 can spread by human-to-human transmission via direct contact or droplets [19–21]. For example, Zhang et al. (2020a) took into account government intervention factors (staying at home, lockdown, isolation and social distance) in their study on the turning point and duration of the COVID-19 epidemic in 6 countries [5]. Zhang et al. (2020b) suggests that the contact rate will affect the dynamics of COVID-19 outbreaks, and that increasing social distance can effectively curb the novel spread of the coronavirus [22]. Their research also shows that China has adopted strong non-drug interventions, including rapid isolation of cases, tracking of close contacts of cases, strict restrictions on the movement and contact of people, and raising awareness of disease and prevention among the population, effectively cutting off the spread of the virus at the community level and making an important contribution to blocking local transmission and spread outside Hubei Province. At present, the introduction of various levels of lockdown requires an adaptation of the typical epidemic measures to this new situation. Some examples relating to the Chinese outbreak can be found in the study of [8, 23]. An Italian study also pointed out that according to the current evolving situation in Italy, restrictions can be measured by introducing a non-constant infection rate [9]. In addition, most existing prediction models use estimated parameters or fixed parameters, which affect the accuracy of evaluation results. It could be argued that the epidemic simulation parameters in different regions and different periods should be a dynamic process.

In response to COVID-19, although the USA government adopted measures such as “lockdown”, “home isolation” and “shelter” in March, due to citizens’ broadly weak awareness of prevention and control, it was difficulty for prevention and control measures to be effective. In June, due to the influence of “riot” factors, the epidemic in the USA entered into its second large-scale outbreak. Therefore, when forecasting and modeling the USA’s COVID-19 epidemic trend, the impact of the policy of social distance and riot outbreak factors on the infection rate should be taken into account.

In view of this, and based on the COVID-19 data and human mobility data of national and state regional administration units in the USA, this paper divides the early change process of COVID-19 in the USA into three stages: initial stage, lockdown period, and riot period. On this basis, a SIRD model was constructed to predict the trend of the epidemic, in order to provide a reference point for clarifying the epidemic spread rule of COVID-19 and promoting the resumption of work and production.

## Materials and methods

### Data source

This study focuses on the epidemic trend of COVID-19 in the national and state regional administration units of the USA (including the District of Columbia, Guam, the Northern Mariana Islands, Puerto Rico and the Virgin Islands). The selected epidemic data are mainly composed of infected cases, recovered cases, and deaths. The data used to observe the spatial transmission of infected cases were taken from the global epidemic real-time monitoring system released by John Hopkins University (https://github.com/CSSEGISandData/COVID-19), which selected period is from January 22 to July 26, 2020. The data for future simulation prediction are based on the real-time dynamic data of COVID-19 cases in American states provided by 1Point3Acres (https://coronavirus.1point3acres.com/en), which covers the relatively complete data pertaining to infected, recovered and fatal cases since March 13, 2020. These data have been cited by a number of authoritative organizations, including the USA Centers for Disease Control and Prevention (CDC), the United Nations (UN) and John Hopkins University (JHU), and thus have a high degree of credibility. The selected data period in this case is March 13 to July 26, 2020.

The human mobility data were obtained from the Google community mobility report (https://www.google.com/covid19/mobility/), which covers more than 120 countries and shows the changes that have taken place in policies aimed at fighting COVID-19. Specifically, the reports charts movement trends over time by geography, across different categories of places such as retail and recreation, groceries and pharmacies, parks, transit stations, workplaces, and residential areas. These individual reports aim to provide insights into what has changed in response to policies aimed at combating COVID-19, and may help to understand how human mobility has impacted on infection rates.

### Model building

**Fig. 1:**
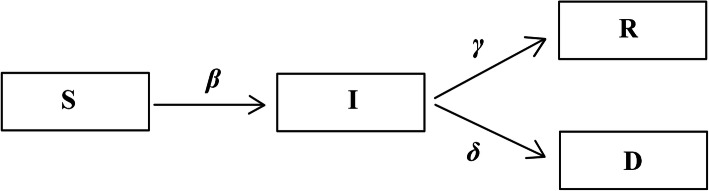
COVID-19 ‘s SIRD epidemic warehouse structure chart

The SIR model divides the population undergoing the epidemic into three categories [11, 12]: Susceptible (refers to a group of residents who are not infected with the disease but lack immunity to the disease), infected (refers to a group of residents who have been infected with the disease and have the ability to spread it), and removed (refers to groups of residents who are immune or who are no longer involved in the spread of the disease due to death, etc.). On the basis of the SIR model, we adjusted the removed (R) population, and increased the death population (D), to form the SIRD model [17, 18]. The SIRD model is an extension of the SIR model. The difference between the two is that the SIRD model subdivides the removed population in the SIR model into the recovered group and the fatality group. This means that the SIRD model divides the resident group into four categories: Susceptible, Infected, Recovered and Deceased. The numbers of susceptible, infected, convalescent and deceased people were expressed by: *S(t), I(t), R(t), D(t)* at *t* moment. Assuming that the total population N remains stable (regardless of birth, death, migration, etc.), then: *S(t) + 1(t) + R(t) + D(t) = N*, where *N* is constant. The structural diagram of COVID-19 ‘s SIRD epidemic chamber is shown in Fig. 1.

The ordinary differential equations (ODEs) of the SIRD model are as follows [17, 18]:

1 $$ \frac{dS}{dt}=\frac{-\beta IS}{N} $$

2 $$ \frac{dI}{dt}=\frac{\beta I S}{N}-\gamma I-\delta I $$

3 $$ \frac{dR}{dt}=\gamma I $$

4 $$ \frac{dD}{dt}=\delta I $$

In the formula, the initial condition is set at t0 time, *[S(t0), I(t0), R(t0), D(t0)] = [S0, I0, R0, D0]*. The parameter *β* is the infection rate, i.e., the probability per unit of time that a susceptible individual will contract the disease when entering into contact with an infected person. The parameters *γ* and *δ* respectively denote the recovery and death rates.

When modeling the epidemic trend of COVID-19, it is necessary to consider the influence of human mobility and social distancing on the infection rate. In line with the current development stage of COVID-19 in the USA, this study also adds in the “lockdown” and “riot” factors. The current epidemic situation of COVID-19 in the USA is divided into three stages (initial period, lockdown period and riot period). The influence of human mobility at different stages of the spread of COVID-19 was subsequently observed based on these divisions, where *β* corresponds to the infection rate in each period [17]. As shown in formula (5):

5 $$ \beta (t)=\left\{\begin{array}{l}{\beta}_0\left( when\kern0.24em t<{t}_{lockdown}\right)\\ {}{\beta}_0\;\exp \left[-\frac{\left(t-{t}_{lockdown}\right)}{\tau_{\beta }}\right]\left( when\kern0.36em {t}_{lockdown}\le t<{t}_{riot}\right)\\ {}{\beta}_0\;\exp \left[-\frac{\left(t-{t}_{lockdown}\right)}{\tau_{\beta}\times {f}_{riot}}\right]\left( when\kern0.24em t\ge \kern0.36em {t}_{riot}\right)\end{array}\right. $$

In this formula, *β*_*0*_ represents the initial infection rate, and *τβ* represents the decay period; *t* is the time node, and *t*_*lockdown*_ is the time node of the lockdown; *t*_*riot*_ is the time point of the riot, and *f*_*riot*_ indicates the spread index caused by the riot.

In order to improve the accuracy of the prediction results, the SIRD model was used to fit the COVID-19 epidemic situation forecast data and the actual data. Regarding ordinary differential equations similar to the SIRD model, this study used a using a bounded trust region (TRF) algorithm to perform non-linear least-squares regression using Python, and determined a 95% confidence interval to optimize the parameters of the ordinary differential equations. In order to verify the accuracy of the model, this study employed the MSLE (Mean Squared Log Error). This index corresponds to the expectation of the square logarithm (quadratic) difference and is applicable to the type of exponential growth examined here. The specific formula is as follows:

6 $$ MSLE=\frac{1}{n}\sum \limits_{i=1}^n{\left[\log \left({Y}_{true}+1\right)-\log \left({Y}_{predict}+1\right)\right]}^2 $$

Here, MSLE represents the root mean square logarithmic error between the actual and predicted data of the epidemic, and *n* represents the number of observations; *Y*_*ture*_ indicates actual epidemic data and *Y*_*predict*_ indicates forecast epidemic data.

## Results

### General epidemic trend of COVID-19 in the USA

#### COVID-19 spatio-temporal differentiation characteristics

**Fig. 2:**
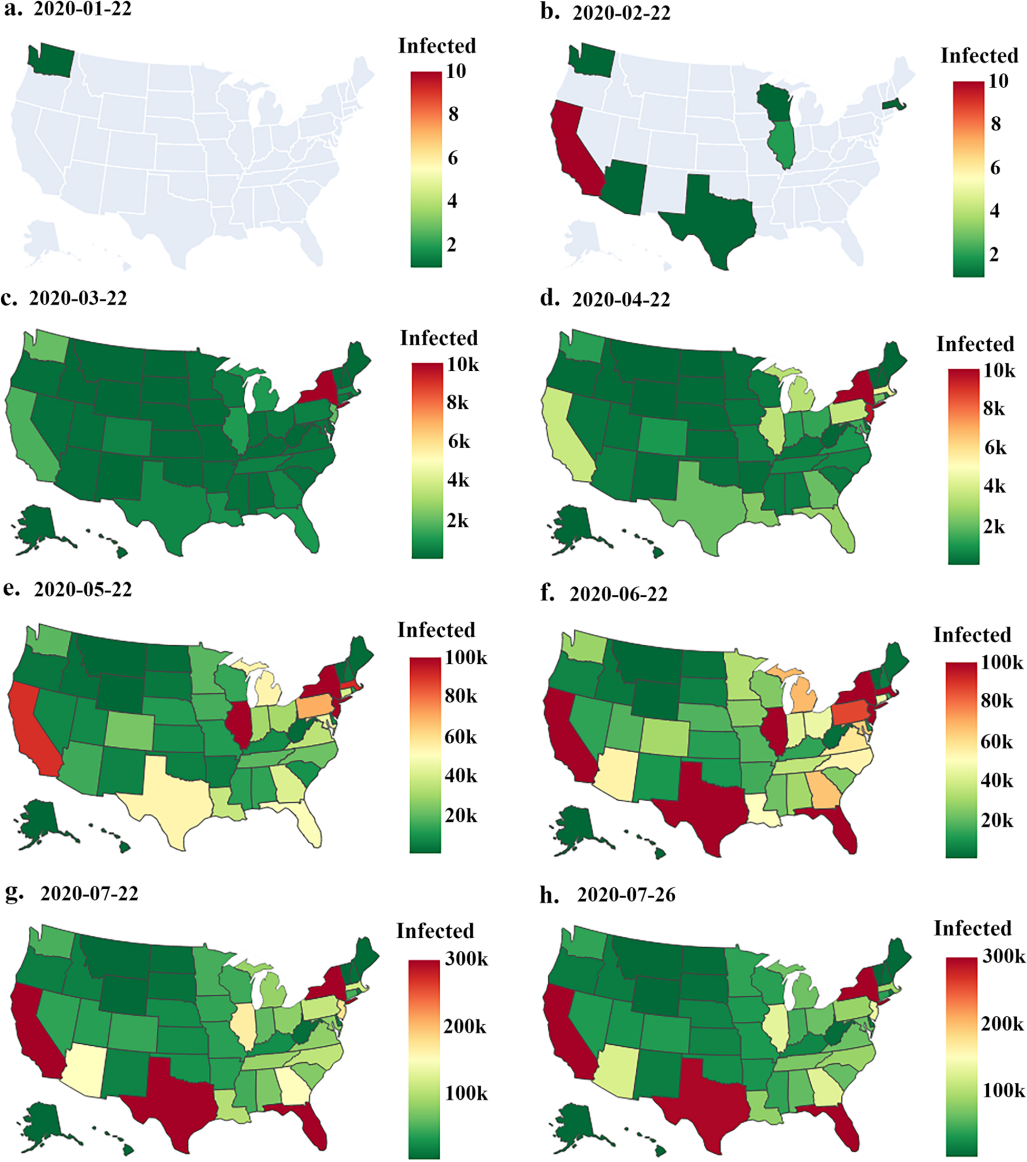
Spatial distribution of cumulative infected cases of COVID-19 in the USA

Based on the collected data pertaining to cumulative infected cases of COVID-19 in the USA, the spatial and temporal evolution trends of COVID-19 from January 22 to July 26, 2020, were drawn using Python (Fig. 2), and the spatio-temporal pattern and evolution characteristics of the early epidemic situation in the USA were analyzed.

As can be seen from Fig. 2, the infected cases of COVID-19 in the USA first appeared in Washington State on January 22nd, while no COVID-19 outbreak was found in other parts of the USA, indicating that this was the beginning of the epidemic in the USA. On 22 February, infected cases of COVID-19 were reported in Washington, Illinois, California, Arizona, Massachusetts, Wisconsin and Texas, and the number of epidemic areas increased from 1 to 7, indicating an expansion of the spatial scope of the epidemic in the USA compared with January 21. It is worth noting that among the seven epidemic states, California announced the largest number of cumulative infected cases, reaching 10, while the other six states reported only 1–2 infected cases, during which period the USA was still in the early epidemic stage. In addition, over time, the spatial spread process of the epidemic shows the characteristics of “geographical proximity”, gradually spreading to neighboring areas with the initial infection areas as the center. On March 22nd, COVID-19 outbreaks occurred to varying degrees in all parts of the USA, especially in New York State. Even though, at this point, USA states successively adopted emergency response measures such as “maintaining social distance” policies to curb the spread of the epidemic, on the whole these measures have not achieved the desired results.

The COVID-19 epidemic situation in the USA continued to deteriorate on April 22nd, with the cumulative number of infected cases exceeding 830,000. The cumulative number of infected cases recorded in New York alone reached 260,000. The reason for this situation may be due to the frequent population movement between different regions, the long latent cycle of COVID-19, and the lack of government screening and testing capacity. On May 22nd, the cumulative infected cases of COVID-19 in various states of the USA further increased, with the number of infected cases exceeding 1.6 million, including more than 100,000 in New York, New Jersey and Illinois, and more than 350,000 in New York. On June 22 ^nd^, the epidemic worsened most significantly in Texas and Florida, with an increase of 50,000 infected cases and a cumulative total of more than 100,000 cases in 1 month. As of July 26, there were more than 4 million infected cases and 140,000 deaths in the USA. The cumulative number of infected cases in California and Florida surpassed that of New York, with California becoming the region with the worst epidemic, and the cumulative number of infected cases in California, Florida, New York and Texas each exceeded 400,000.

#### Analysis of the correlation between human mobility and the COVID-19 epidemic

**Fig. 3:**
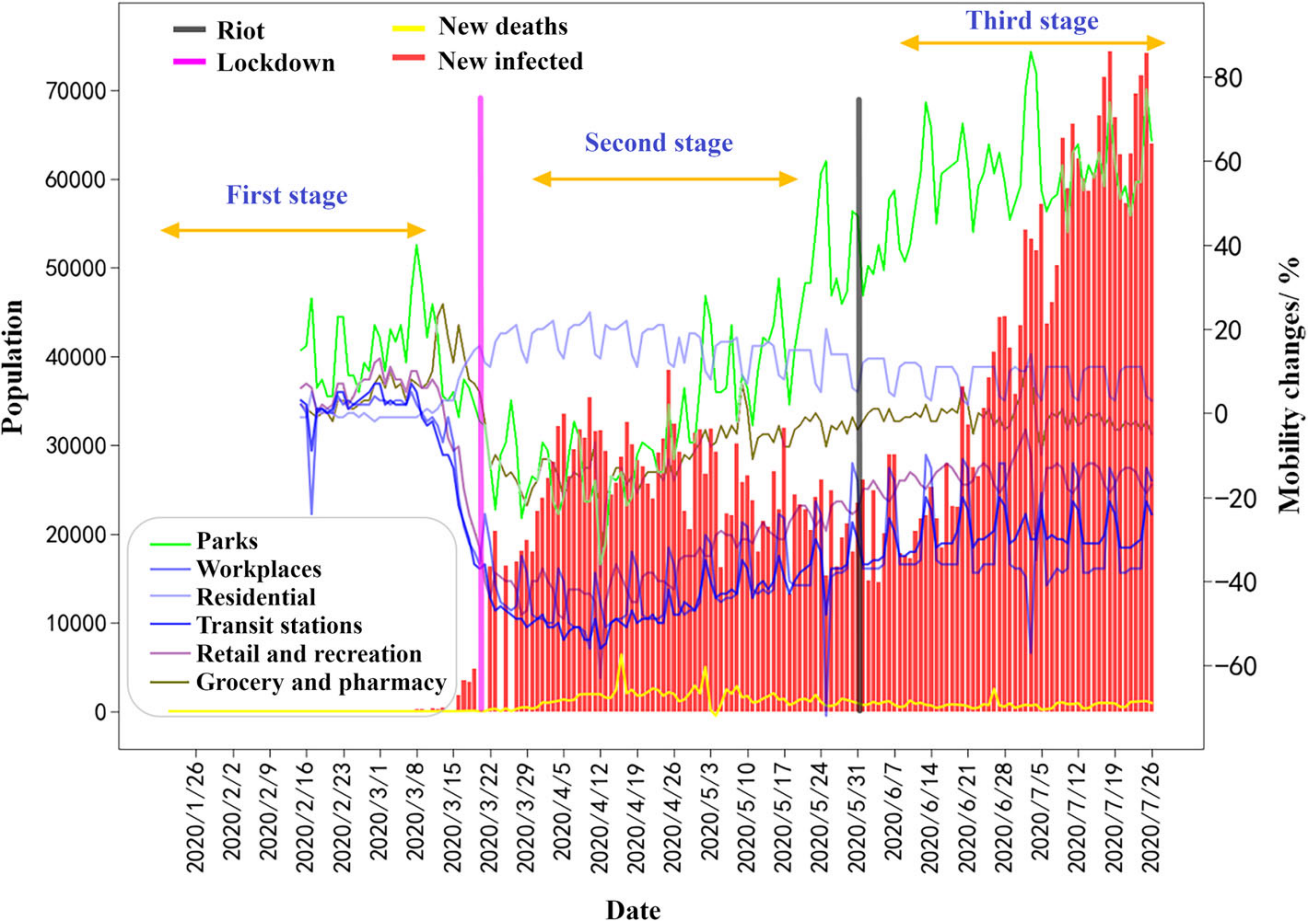
Daily trends in human mobility and COVID-19 cases (residential mobility of 15% represents a 15% change in visits to places of residence compared to baseline)

In this section, we use Google’s mobility data to analyze how the mobility of American residents changed during quarantine (Fig. 3). Given that the incubation period of the virus driving this epidemic is between 1 to 14 days, there is a certain time lag in the data [24]. Furthermore, the lag time is observed by Pearson correlation, which was here used as the real correlation index.

As can be seen from Fig. 3, the number of COVID-19 infections in the USA from January to February 2020 is relatively low. The reason is that COVID-19 outbreaks were found in only a few areas such as New York, Washington and Vermont during this period. At the time, people were not paying enough attention to the epidemic, and a large number of virus carriers were not screened out in a timely and effective manner. These aspects, coupled with the weak intervention measures taken by the government, meant that the epidemic evolved to a state of free spread (First stage).

Since the USA government adopted measures such as “lockdown”, “home isolation” and “shelter” in March 2020, the number of residents’ home activities increased, while other outbound activities showed a significant downward trend. Unfortunately, due to citizens’ generally weak awareness of prevention and control, the effectiveness of the latter measures was limited. On the one hand, with the improvement of testing ability, increasing numbers of people were diagnosed as positive. On the other hand, American citizens’ pursuit of “individuality” and “yearning for freedom”, not wanting to be “bound”, makes them resistant to the policy of maintaining a social distance. In addition, the government encouraged “restart” and “return to work and production” as soon as possible, and even permitted open bars and other places of mass entertainment to remain open. Around the same time, with the warming of the weather, residents’ outdoor activities increased significantly in May, and the number of stays in the resident began to decrease. For example, during the lockdown, many residents still engaged in recreational activities without wearing masks. They thought the epidemic was just a common flu, and that news reports had exaggerated the spread and death rates of the epidemic (https://www.foxnews.com/health/coronavirus-parties-washington-state-county-rise-in-cases). During this period, the epidemic in the USA actually entered the stage of large-scale outbreak and, in a sense, acquired a state of semi-free transmission (Second stage).

On May 25, a black man in Minnesota was suffocated to death by the violent law enforcement of white police, which shocked the USA. On that day, demonstrations began in Minnesota and gradually developed into riots (https://www.sohu.com/subject/320511). Since the beginning of June, as the “riots” have continued to escalate (https://www.sohu.com), large-scale protests have been held in 22 states and 140 cities in the USA, leading to the USA’s second outbreak of COVID-19 (Third stage) .

**Fig. 4:**
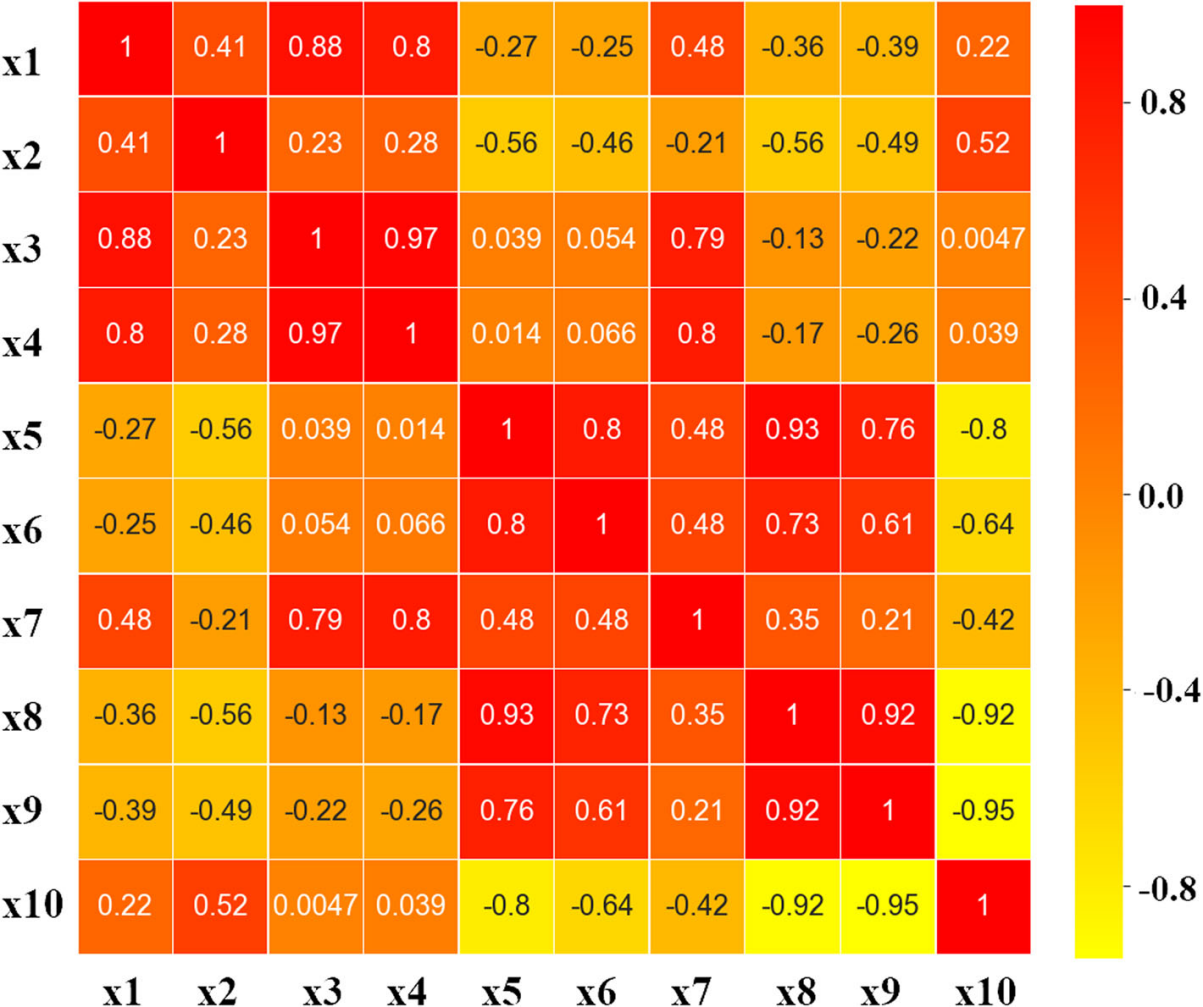
Heat map of the correlation between human mobility and COVID-19 (X1: New confirmed, X2: New deceased, X3: Total confirmed, X4: Total deceased, X5: Retail and recreation, X6: Grocery and pharmacy, X7: Parks, X8: Transit stations, X9: Workplaces, X10: Residential areas)

Figure 4 highlights the Pearson correlation between the COVID-19 pandemic and the related indicators of human mobility. Among the many outdoor activities, a significant positive correlation can be seen between the number of new infected cases and the number of residents visiting parks, there is a particularly strong positive correlation between the daily cumulative number of infected cases and such park visits, as high as 0.79 (*p* < 0.01). Therefore, when forecasting and modeling the USA’s COVID-19 epidemic trend, the impact of social policy, social distancing and riot outbreak factors on the infection rate should be taken into account.

### Prediction and analysis of the future trend of COVID-19 in the USA

Based on the above analysis, it can be seen that in the first stage, there was insufficient understanding of the epidemic, when its spread was in the stage of free growth. The second stage is that of blockade due to the lockdown measures taken by the government, which greatly reduced human mobility. Given that the per capita contact rate per unit of time will show an exponential downward trend, the infection rate *β* in the study is described by an exponential function. The third stage encompasses the large-scale demonstrations triggered by the “riots” in the USA, which makes the contact rate rise sharply over a short period of time and marks the second growth stage; thus, here, increasing riot factors are considered in order to adjust the infection rate *β*. The transmission index *f*_*riot*_ caused by the riots is assumed to have a great impact on the spread of the epidemic in the USA. After repeated verification of the model, the value of *f*_*riot*_ is: *1 ≤ f*_*riot*_ *≤ 3*. On this basis, the *f*_*riot*_ in formula (5) was adjusted in order to better predict the future trends.

#### Prediction and analysis of future trends at national level

**Fig. 5:**
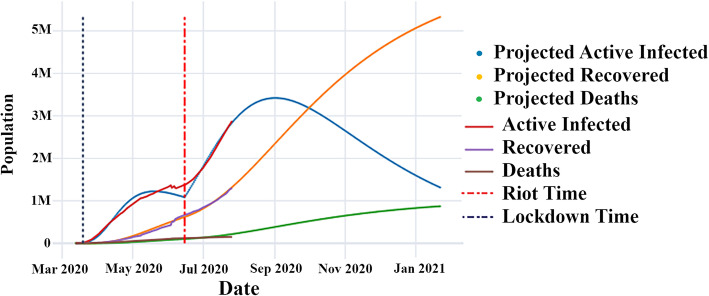
Predicted evolution of the COVID-19 outbreak in the USA

This study is based on 1Point3Acres data on infected, recovered and fatal cases of COVID-19 at the national and state scales of the USA from March 13 to July 26, 2020. After constructing the dynamic model of SIRD infectious diseases, we used Python software to simulate the trend of the epidemic in the USA as a whole within a certain future time period. The predicted results are shown in Fig. 5.

Observing the change curve of the number of simulated infections, it was found that the turning point of the epidemic appeared on September (Fig. 5). At the moment of this turning point, our model predicts that the cumulative number of infected cases in the USA will reach 6,144,748, the stock of active infected cases will reach 3,419,974, the cumulative number of recovered cases will reach 2,341,461, and the cumulative number of deaths will reach 383,313 (Table 1). From the turning point moment to the end of the model forecast (January 22, 2021), the number of COVID-19 infections in the USA will continue to decline; in October, the cumulative number of cured people will exceed the stock of active infected cases, and the number of cured people will usher in a large increase. At the end of the forecast period, the final total number of infected cases will reach 7,511,775, the stock of active infected cases will remain at 1314529, the cumulative number of recovered cases will remain at 5325488, and the cumulative number of deaths will remain at 871758. The cumulative number of infected cases is set to be huge, exceeding 7.51 million, which is bound to have a great impact on the social economy of the USA. The government of the USA should thus prepare for a long-term response to the epidemic and take strict control measures to reduce the number of residents going out as much as possible.

**Table 1:** Case size at turning points and projected end period

	Turning point					Prediction end (2021/1/22)				
	Date	Infected	Active	Recovered	Deaths	Date	Infected	Active	Recovered	Deaths
**USA**	2020/9/01	6,144,748	3,419,974	2,341,461	383,313	2021/1/22	7,511,775	1,314,529	5,325,488	871,758
**CA**	2020/9/21	873,409	438,786	385,440	49,183	2021/1/22	1,153,455	195,246	849,780	108,429
**FL**	–	–	–	–	–	2021/1/22	2,703,855	2,519,370	182,977	1508
**NY**	2020/6/30	404,622	317,700	54,227	32,695	2021/1/22	419,225	218,496	123,759	76,970
**TX**	2020/8/28	772,034	224,670	20,412	526,952	2021/1/22	1,189,513	8770	45,561	1,176,187
**NJ**	2020/7/07	176,512	155,455	4492	16,565	2021/1/22	669,051	112,960	14,681	541,410
**IL**	2020/8/10	162,055	150,185	–	11,870	2021/1/22	460,436	132,583	–	327,853
**GA**	2020/12/18	595,363	477,029	60,980	112,236	2021/1/22	687,319	466,026	77,905	143,388
**AZ**	–	–	–	–		2021/1/22	548,245	482,318	58,993	6934
**MA**	2020/7/15	113,565	103,278	–	10,287	2021/1/22	116,428	82,783	–	33,645
**PA**	2020/8/06	108,062	94,315	2504	11,243	2021/1/22	112,056	74,588	6824	30,644

Based on the MSLE error of the model (Table 2), the MSLE error of the stock of active infected cases in the USA is only 0.0145, the MSLE error of cumulative recovered cases is 0.0716, and the MSLE error of cumulative death cases is 0.5293, indicating that the SIRD model constructed in this study can well fit the change curve of cumulative recovered cases in the USA. In addition, by observing Fig. 3, it emerges that the change curve of the actual number of recovered cases in the study period shows a fluctuating and rising trend, and that the number of recovered cases will see a relatively large increase.

**Table 2:** Partial parameter settings and MSLE error results

	***β***_***0***_	***τβ***	γ	***δ***	Active MSLE	Recovered MSLE	Deaths MSLE
**USA**	0.2965 (0.2952, 02978)	18.2532 (18.1289, 18.3776)	0.0085 (0.0083, 0.0088)	0.0014 (0.0012, 0.0016)	0.0145	0.0716	0.5293
**CA**	0.1408 (0.1378, 0.1438)	52.4868 (51.1321, 53.8414)	0.0135 (0.0128, 0.0143)	0.0021 (0.0015, 0.0027)	0.4805	1.0055	0.8436
**FL**	0.1457 (0.1369, 0.1404)	45.5202 (44.0674, 46.973)	0.0000 (0.0000, 0.0005)	0.0006 (0.0000, 0.0011)	1.3653	12.1147	3.9553
**NY**	0.1450 (0.1377, 0.1523)	18.0593 (17.6187, 18.4999)	0.0013 (0.0012, 0.0013)	0.0008 (0.0007, 0.0008)	0.0002	0.0024	0.0291
**TX**	0.2252 (0.2236, 0.2269)	38.2234 (37.2958, 39,151)	0.0022 (0.0017, 0.0028)	0.0469 (0.0457, 0.0481)	0.3292	0.5997	0.7387
**NJ**	0.3336 (0.3278, 0.3394)	16.5512 (16.0440, 17.058)	0.0010 (0.0007, 0.0012)	0.0022 (0.0019, 0.0024)	0.1107	0.8469	0.5487
**IL**	0.2649 (0.2622, 0.2660)	22.8471 (22.5580, 23.1362)	–	0.0009 (0.0008, 0.0010)	0.0646	–	1.1597
**GA**	0.1576 (0.1549, 0.1602)	41.2431 (40.1865, 42.2998)	0.0012 (0.0006, 0.0019)	0.0020 (0.0014, 0.0027)	0.8875	2.9482	1.9281
**AZ**	0.2092 (0.2082, 0.2103)	18.1688 (18.0336, 18.3040)	0.0008 (0.0006, 0.0010)	0.0001 (0.0000, 0.0002)	0.1260	1.1917	0.8466
**MA**	0.2332 (0.2325, 0.2340)	18.2453 (18.1355, 18.3550)	–	0.0013 (0.0013, 0.0014)	0.0118	–	0.4421
**PA**	0.2976 (0.2964, 0.2987)	15.4270 (15.3304, 15.5235)	0.0003 (0.0002, 0.0004)	0.0013 (0.0012, 0.0015)	0.0053	1.0329	0.3800

The latter finding shows that in the short term, the cumulative number of recovered cases in a certain place is closely related to the local epidemic prevention and control measures, and to medical and health conditions. However, in the long run, with the continuous improvement of epidemic prevention and control measures, the cumulative number of recovered cases shows momentum in terms of rapid growth, especially in late May. The intersection of the fitting curve between the number of infected and the number of recovered cases appears in early October, implying that the COVID-19 epidemic in the USA is expected to be brought under control from this time period.

#### Prediction and analysis of future trends at state level

**Fig. 6:**
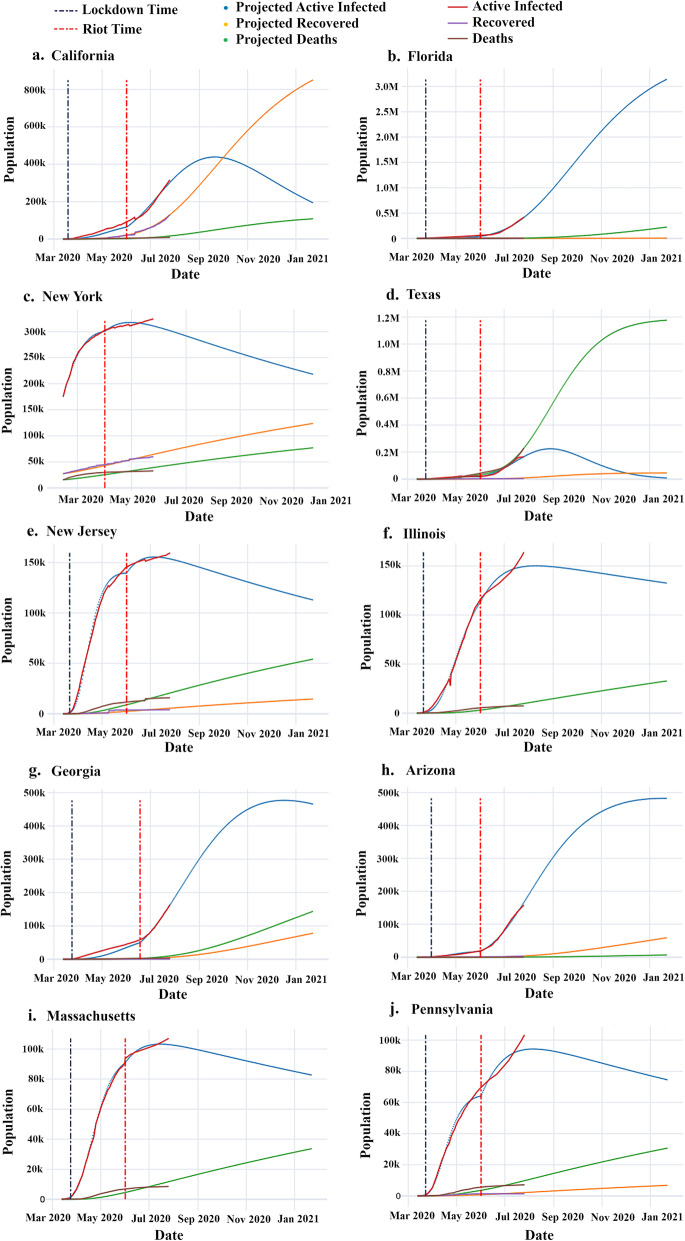
Forecast curve of COVID-19 epidemic in severely affected areas of the USA

For convenience of analysis, this study selected 10 states with a serious number of infected cases in the USA on July 26, 2020 (including California, Florida, New York, Texas, New Jersey, Illinois, Georgia, Arizona, Massachusetts and Pennsylvania), on which to focus the analysis (Fig. 6). The forecast period t is 180 days (January 22, 2021), based on which the epidemic trend of the regional administrative unit at the state level is discussed. It should be noted that due to the COVID-19 data of various states having been repeated or not being recorded for several consecutive days, this may have led to large errors in the prediction results. In order to ensure the smooth implementation of the study, for those states with large fluctuations in a certain period of data in the study, such as New York, the data after April 14 were selected as the observation data. For states with large fluctuations in recovered cases data, such as Illinois and Massachusetts, only infected and fatal cases were simulated. The predicted future trend is shown in Fig. 6, and the scale of the epidemic at the turning point and the end of the forecast period are shown in Table 1.

As can be seen from Fig. 6, New York, New Jersey and Massachusetts reached the epidemic turning point in July during the 180-day prediction process. By then, the cumulative number of infected cases in New York, New Jersey and Massachusetts were 404,622, 176,512 and 113,565, respectively, and the cumulative number of deaths was 32,695, 16,565 and 10,287, respectively. Texas, Illinois and Pennsylvania are predicted to reach the turning point of the epidemic in August, when the cumulative number of infected cases in Texas, Illinois and Pennsylvania will be 772,034, 162,055 and 108,062, respectively, and the cumulative number of deaths 526,952, 11,870 and 11,243, respectively. California and Georgia are forecast to hit the turning point in late September and mid-December, respectively. In October, the cumulative number of recovered cases in California will exceed the number of active infected cases, and the number of recovered cases will enter the stage of rapid growth, while the number of active infected cases will enter the stage of decline. Georgia will have a big increase before it reaches the turning point, and government departments should continue to take strict control measures during this period. At that time, the cumulative number of infected cases in California and Georgia will be 873,409 and 595,363 respectively, the cumulative recovered cases 385,440 and 60,980 respectively, and the cumulative deaths cases are 49,183 and 112,236, respectively. It should be noted that by the end of the forecast period, Florida and Arizona will have failed to reach the turning point of the epidemic, and the number of infected cases there will still show a rapid growth trend, indicating the worsening of the epidemic in these states. Taking more stringent control measures to slow down the spread of the epidemic is thus an urgent task for these areas.

#### Diagnosis and SIRD model test

On the Python platform, the bounded trust region (TRF) algorithm was used to perform a non-linear least-squares simulation to simulate the epidemic trend of different states. This enabled us to obtain the fitting parameters of different regions and their 95% confidence intervals (CI), based on which we could subsequently diagnose and test the SIRD model in each region (Table 2).

In order to effectively contain the spread of the COVID-19 epidemic, the states of the USA have taken a series of emergency prevention and control measures. However, as the infected, recovered and death cases in each state remain in a dynamic process, significant differences have also emerged in the epidemic prevention and control measures taken by the different states, and the conditions of local medical and health facilities. All of these mean that if the unified threshold parameters (including infection rate, recovery rate, death rate, etc.) are used in the construction of SIRD model, this is bound to increase the cumulative error to some extent, thus affecting the accuracy of the SRD model’s prediction results.

In view of this, the parameters adopted in this study were not fixed, but adaptive dynamic parameters in the process of epidemic trend prediction. According to Table 1, the initial infection rate *β0* value, decay period *τβ* value, recovery rate *γ* value and death rate *δ* value of the USA were 0.2965, 18.2532, 0.0085 and 0.0014, respectively.

Among the 10 regions seriously affected by the COVID-19 epidemic in the USA, California can be seen to have the highest recovery rate, with a *γ* value of 0.0135. The recovery rates in Texas, New York and Georgia are 0.0022, 0.0013 and 0.0012, respectively. The recovery rates in Arizona and Pennsylvania are in the medium level, with *γ* values of 0.0008 and 0.0003, respectively. Florida and New Jersey have the lowest recovery rates. In terms of the death rate, Texas can be seen to have the highest value, with a *δ* of 0.0469. New Jersey, California and Georgia also have high death rates, with respective *δ* values of 0.0022, 0.0021 and 0.0020. The death rate rates in Massachusetts and Pennsylvania are at the medium level, with a *δ* value of 0.0013 for both. Florida, New York and Illinois are at low levels, with *δ* values between 0.0006–0.0009. Arizona has the lowest death rate, with a *δ* value of only 0.0001.

By observing the MSLE error between the predicted and actual active infected cases change curve, it can be seen that Florida and Georgia have the largest MSLE error, while New York, Massachusetts, Pennsylvania, Illinois, New Jersey and Arizona have relatively small MSLE errors. The key factor causing the deviation between the simulated value and the actual value may be due to the repeatability and lag of data reporting, resulting in a substantial increase or decrease in the number of cases within a certain day.

## Conclusion and discussion

Based on COVID-19 epidemic data and human mobility data in the USA from January 22 to July 26, this study uses the improved SIRD model to predict early epidemic trends in national and state regional administration units in the USA from July 27, 2020, to January 22, 2021. The conclusions are as follows.

1. There are spatio-temporal differences in the COVID-19 epidemic across the USA. In terms of temporal changes, the current spread process of the epidemic in the USA can be divided into early outbreaks, large-scale outbreaks and slow recession stages, with the implication that the epidemic will not end in a short period of time. In terms of spatial distribution, the worst-hit areas are mainly located in the northeastern USA and areas along the Great Lakes, with sporadic distribution in the southwest, southeast and south of the country.
2. The epidemic situation in various states of the USA shows similar characteristics of phased changes, while the epidemic trajectory in other areas shows certain peculiarities. The development of the epidemic in the 10 most seriously affected regions can be divided into three stages: early outbreak, large-scale outbreak and slow recession; however, the turning point of the epidemic will be different in different regions.
3. There is a strong correlation between the number of infected COVID-19 cases and the activities of residents visiting parks. Due to the impact of riots, the frequency of residents visiting parks greatly increased the infection rate of the epidemic, resulting in varying degrees of postponement of the turning points in the USA as a whole, and also in specific states.
4. The turning points of the epidemic in the USA at the national and state levels show a high degree of consistency. Specifically, the turning point of the epidemic in the USA as a whole is predicted to occur on September and among the 10 states with experiencing the most severe epidemic, New York, New Jersey, Massachusetts, Texas, Illinois, Pennsylvania and California are all to meet this point in a concentrated period from July to September.

Based on the above conclusions, it is suggested that the USA government should adopt the lessons learned from the epidemic prevention and control measures applied in some areas, scientifically divide risk prevention and control areas, implement hierarchical management and control, and promote the resumption of work and production in an orderly manner, so as to avoid the negative impact of excessive epidemic prevention on society and people’s livelihood. At the same time, measures such as quarantine, isolation and psychological counseling should be taken to encourage people to maintain social distancing and avoid group activities. With regard to individual residents, it is recommended that they enhance their awareness of relevant precautions, avoid taking public transportation, and avoid going to closed places where crowds gather. In addition, when symptoms such as fever, cough and vomiting occur, they should self-isolate and report their symptoms, and pay attention to developing good personal hygiene habits, such as more ventilation, washing their hands frequently, wearing masks and not facing others while sneezing, etc. Autumn and winter are high seasons for the spread of epidemics and, as no vaccination for COVID-19 has yet been developed, apart from personal hygiene, the only way to control the spread is for every citizen to strictly abide by lockdown to prevent the virus from rebounding again in the USA before reaching the turning point.

This study predicts the epidemic trends of COVID-19 in the USA by constructing a SIRD model and considering “social factors”. The aim here is to provide a reference for clarifying the epidemic spread rule, scientifically formulating an epidemic prevention and control plan, and for promoting the resumption of work and production in an orderly manner. However, this study has the following shortcomings. First of all, due to the large fluctuations or absence in the recovered case data of residents in some states, there will inevitably be a big deviation between the predicted results and the actual data. Second, the spread in the early outbreak stage of the epidemic was more of a state of free transmission. With the increasing attention of the government, society and residents paid to the epidemic, many temporary control measures have been introduced; in this scenario, the free spread of the virus is difficult to maintain. Although the study divides the spread process of the epidemic in the USA into stages according to government intervention measures and social emergencies, it is necessary to acknowledge the complexity of real social activities, which complicates the positing of accurate mathematical expressions and predictions. Therefore, there is uncertainty in using this model to predict the epidemic trend in the future under different intervention intensities and emergencies. How to extend the prediction period while ensuring the accuracy of prediction is a key direction for future research. Third, due to the limitations of objective factors such as virus incubation period, detection capability, and medical and health facilities, the current official epidemic data may be lower than the actual infection data, which will also lead to uncertainty in the final evaluation results. Finally, this study only predicts the future epidemic trend at the national level and in some state administrative units in the USA, and does not put forward effective prevention and control measures, thus restricting the research depth and application value of this paper.

## Data Availability

The datasets used and/or analyzed during the current study are available from the corresponding author on reasonable request.
